# Lectin Histochemistry of the Normal Feline Kidney

**DOI:** 10.3390/vetsci10010026

**Published:** 2022-12-30

**Authors:** Ayana Noguchi, Natsume Kurahara, Osamu Yamato, Osamu Ichii, Akira Yabuki

**Affiliations:** 1 Laboratory of Veterinary Clinical Pathology, Joint Faculty of Veterinary Medicine, Kagoshima University, 1-21-24 Korimoto, Kagoshima 890-0065, Japan; 2 Laboratory of Anatomy, Department of Basic Veterinary Sciences, Faculty of Veterinary Medicine, Hokkaido University, Sapporo 060-0818, Japan; 3 Laboratory of Agrobiomedical Science, Faculty of Agriculture, Hokkaido University, Sapporo 060-8589, Japan; 4 Kagoshima University Veterinary Teaching Hospital, Joint Faculty of Veterinary Medicine, Kagoshima University, 1-21-24 Korimoto, Kagoshima 890-0065, Japan

**Keywords:** cat, kidney, lectin histochemistry, nephron segment

## Abstract

**Simple Summary:**

Cats are highly susceptible to chronic kidney disease (CKD); however, the pathophysiological mechanisms of feline CKD have not been fully elucidated. To understand the mechanisms of CKD, it is important to clarify the changes in glycosylation of diseased renal cells. Lectins are specific probes used to explore changes in the carbohydrate moiety of cellular components. No study has yet demonstrated lectin binding patterns in normal feline kidneys. In the present study, we performed a lectin histochemical analysis of normal feline kidneys and evaluated the lectin-binding pattern in each nephron segment. Eight lectins, WGA, s-WGA, RCA-I, ConA, PNA, SBA, DBA, and UEA-I, were used as probes in the present study. The lectin binding pattern in each nephron segment was determined for each lectin, and a feline-specific lectin-binding pattern was established in the normal kidney. The present study provides a basis for future glycopathological studies of feline CKD.

**Abstract:**

Lectins have a strict binding specificity to carbohydrate moieties of cellular components, and can thus indicate changes in the glycosylation of cells in diseases. However, lectin-binding patterns in nephron segments of feline kidneys have not been fully surveyed. The present study reported lectin-binding patterns in normal feline kidneys by histochemical investigations of eight commercially available lectin detection kits. Kidneys from four normal cats (intact males, 23–27 months old) were fixed in 4% paraformaldehyde, and embedded in paraffin; lectin histochemistry was performed for WGA, s-WGA, RCA-I, ConA, PNA, SBA, DBA, and UEA-I lectins. WGA, RCA, and ConA binding was observed from Bowman’s capsule to the collecting ducts, while only WGA was detected in the glomerular capillary. s-WGA was observed from the proximal tubules to the collecting ducts, showing discriminative heterogeneous binding. PNA and SBA were detected in the distal nephrons, such as the thin limbs of the loops of Henle, distal tubules, and collecting ducts. UEA-I binding was observed in the thick ascending limbs of the loops of Henle, especially in the macula densa regions. DBA lectin showed no positive labeling in nephrons. The observed binding patterns may prove beneficial for the analysis of changes in glycosylation in feline kidney diseases.

## 1. Introduction

Lectins are proteins with a specific binding activity to sugar moieties on proteins or glycoproteins in plants, animals, and microorganisms [1], which can be used as histochemical probes for identifying residual sugars on cell surfaces and intracellular organelles [2,3,4]. Histochemical studies, performed in the kidneys of humans [3,5,6] and experimental rodents [7,8,9,10], have shown that lectins display strict binding specificity to the cellular components of each nephron segment and can be used as useful probes for identifying nephron segments. These probe properties of lectins have been harnessed to identify the nephron segment of urinary nucleated cells or damaged tubular compartments [11,12,13]. Furthermore, changes in the lectin binding pattern of renal tissues have been investigated as an important renal pathophysiological phenomenon in humans [14,15,16,17] and rodents [18,19], as the carbohydrates of the cell membrane and organelles play important roles in maintaining cell function.

Only a few studies have investigated the lectin histochemistry of the kidneys in small animals. In our previous study, we surveyed normal canine kidneys using lectin histochemistry and clarified the canine-specific lectin binding patterns in the nephron segment [20]. In addition, we surveyed the changes in the binding patterns of kidneys with immune complex glomerulonephritis (ICGN), as one of the major causes of chronic kidney disease (CKD) in dogs, and suggested the pathophysiological diversity of ICGN from the binding pattern [21].

Cats are known to have a high prevalence of CKD, with it being one of the most common diseases in older cats, with increasing prevalence in recent decades [22,23]. CKD in cats is pathophysiologically distinct from CKD in dogs. For example, proteinuria, which is common in canine CKD, is a minor clinicopathological event in feline CKD [24,25]. Tubulointerstitial fibrosis is the major pathological change observed in feline CKD, whereas glomerulonephropathy is the hallmark of canine CKD [26,27,28]. Clarifying the feline-specific pathophysiological mechanisms in the kidneys is of utmost importance for developing a new strategy for the management and prevention of feline CKD, and lectins could be a useful tool to explore the functional changes in each nephron segment during CKD.

However, in cats, lectin histochemical studies have not been conducted on kidney diseases, although lectin binding patterns in the kidneys, with lysosomal storage disease [29] and polycystic kidney disease [30], have previously been reported. Clarifying the lectin-binding pattern in normal feline kidneys is necessary to establishing fundamental data regarding the surveyed pathophysiology of kidney disease in cats; however, the patterns in normal cat kidneys have not yet been fully surveyed, with only one study providing a brief description of lectin binding as findings in control cats [29]. Therefore, in the present study, we surveyed the lectin-binding patterns in normal feline kidneys as clarified by histochemical investigation with eight commercially available lectins.

## 2. Materials and Methods

Kidney samples were collected from four normal cats (intact males, 23–27 months old) obtained from the Hokkaido University, Hokkaido, Japan. These cats had been purchased from Kitayama Labes Co., Ltd., Japan, and used as normal controls for the other study, performed in adherence with the AAALAC International standards. The health of each cat was confirmed by physical examination, with the complete blood count and blood biochemical analysis performed by veterinarians. The collected kidneys were sliced to a thickness of approximately 5 mm (approximately 1 cm × 1 cm), fixed in 4% paraformaldehyde in phosphate buffer at 4 °C for 24 h, embedded in paraffin, and prepared in 3-μm thickness sections. For general histological observations, sections were stained with hematoxylin-eosin (HE), periodic acid-Schiff (PAS), and Masson’s trichrome (MT) stains.

Lectin histochemistry was performed according to the methods described in our previous study [31]. Information regarding the biotinylated lectins (Lectin Kit I and succinylated wheat germ agglutinin; Vector Laboratories, Burlingame, CA, USA) used in the present analysis is summarized in Table 1. Sections were deparaffinized, rehydrated, and treated with 3% H_2_O_2_ for 30 min. After washing in 10 mM phosphate-buffered saline (PBS, pH 7.4), the sections were blocked with 0.25% casein in PBS for 60 min, and then incubated with biotinylated lectins diluted in blocking solution overnight at 4 °C. Subsequently, the sections were incubated with horseradish peroxidase-conjugated streptavidin (ready-to-use; Vector Laboratories) for 30 min after washing with PBS. Lectin-binding labeling was detected using a 3,3′-diaminobenzidine (DAB) system (DAB Buffer tablet, Merck, Darmstadt, Germany) and terminated with cold distilled water. Sections were counterstained with Mayer’s hematoxylin. In the negative control, lectins were replaced with a blocking solution. The specificity of each lectin was confirmed by pre-incubation with the appropriate hapten sugars at 37 °C for 60 min, according to the manufacturer’s instructions (Table 1).

## 3. Results

**Figure 1:**
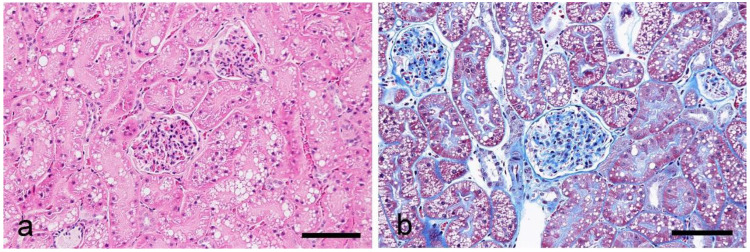
Histological findings of the cortex in the feline kidney. (**a**) HE stain, (**b**) MT stain. Scale bars 100 μm.

No lesions or postmortem changes were observed in any of the kidney tissues in histological observations by HE, PAS, and MT staining (Figure 1). The lectin histochemical findings are summarized in Table 2. No positive labeling was observed in the negative control sections, and lectin labeling was not detected in the sections pre-incubated with hapten sugar.

**Figure 2:**
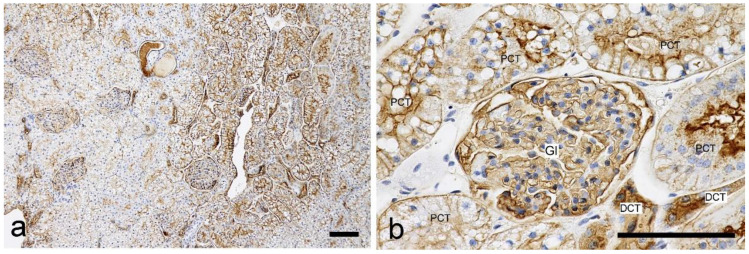
Histochemical staining for wheat germ agglutin (WGA) in the feline kidney. (**a**) Low magnification view of the cortex. Staining intensity is different between areas within the cortex. (**b**) High magnification view of the cortex with positive staining. Gl: glomerulus. PCT: proximal convoluted tubule. DCT: distal convoluted tubule. Scale bars: 100 μm.

**Figure 3:**
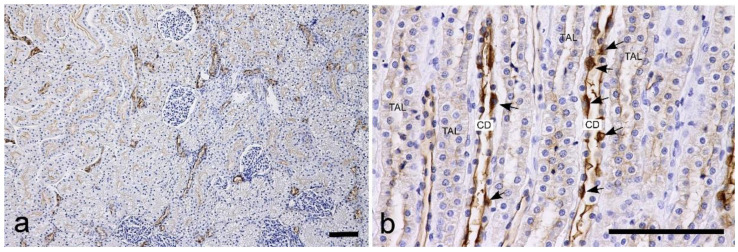
Histochemical staining for succinylated wheat germ agglutin (s-WGA) in the feline kidney. (**a**) Low magnification view of the cortex. The staining intensity of the brush border of the proximal tubules is different between areas within the cortex. (**b**) High magnification view of the inner medulla. TAL: thick ascending limb of the loop of Henle. CD: collecting duct. Arrows: CD cells with positive cytoplasm. Scale bars: 100 μm.

WGA: Positive labeling was detected in a wide range of nephron compartments from the glomeruli to the collecting ducts (Figure 2). In the glomeruli, positive labeling was observed in the external walls of Bowman’s capsules and capillary walls. The proximal tubules (PTs) showed positive labeling in the cytoplasm, brush border, and basement membrane. The labeling for the proximal convoluted tubules (PCTs) and proximal straight tubules (PSTs) were similar. Labeling in the cytoplasm was detected both granularly and sparsely, but was observed only heterogeneously in the glomeruli and proximal tubules. Briefly, the staining intensity in these nephron segments was not consistent throughout the section, with differences between different areas; areas with strong labeling and those with weak labeling were mixed and radically arranged within the same section. The thin limbs (TLs) and thick ascending limbs (TALs) of the loops of Henle, distal convoluted tubules (DCTs), and collecting ducts (CDs), showed strong positive labeling, especially on the apical surfaces.

s-WGA: Positive labeling was widely detected in PTs, TLs, TALs, DCTs, and CDs, but not in the glomeruli (Figure 3). The proximal tubules were positively labelled in the brush border; however, the labeling intensity was heterogeneous, and areas with strong labeling and those with weak labeling were mixed within the same section, as was the case with WGA. Strong labeling was detected on the apical surface in the loops of Henle, distal tubules, and collecting ducts. In the cortical collecting ducts (CCDs) and outer medullary collecting ducts (OMCDs), the cytoplasm showed heterogeneous staining. Briefly, most cells had a negative cytoplasm, but some cells in the same duct were positive (Figure 3). Heterogeneous staining was not observed in the inner medullary collecting ducts (IMCDs).

**Figure 4:**
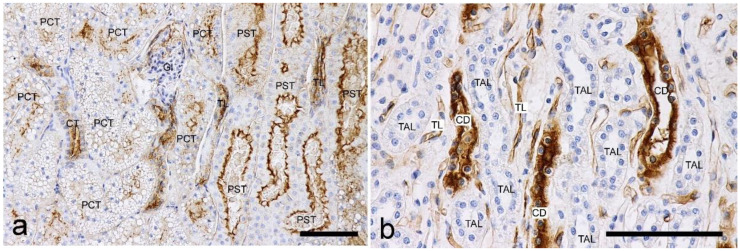
Histochemical staining for *Ricinus communis* agglutin I (RCA-I) in the feline kidney. (**a**) Low magnification view of the corticomedullary junction. A stronger staining intensity can be observed in the brush border of the proximal straight tubules (PSTs) than those of the proximal convoluted tubules (PCTs). (**b**) High magnification view of the inner medulla. Positive labelings are seen in the CDs and thin limbs (TLs). Scale bars: 100 μm.

RCA-I: Clear labeling was observed at the brush borders of the PTs, and the labeling intensity was stronger in the PSTs than in the PCTs (Figure 4). Strong labeling was also observed in the TLs and CDs; however, the TALs of the loops of Henle were negative.

**Figure 5:**
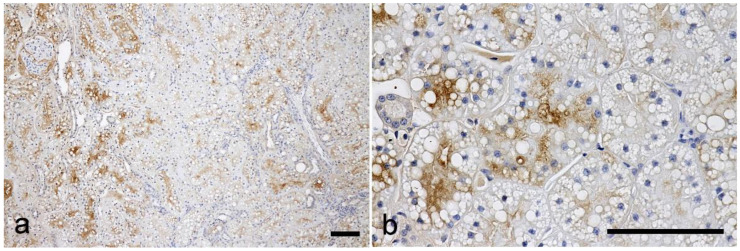
Histochemical staining for concanavalin A (ConA) in the feline kidney. (**a**) Low magnification view of the cortex. (**b**) High magnification view of the cortex. Heterogeneous staining intensities are observed in the proximal convoluted tubules. Scale bars: 100 μm.

ConA: Clear labeling was observed in PTs, especially PCTs. The brush borders and cytoplasm of this segment showed positive labeling; however, the labeling showed clear heterogeneity (Figure 5). Briefly, PCTS in some areas showed stronger labeling, while other areas in the same section showed negative or faint labeling, and areas with different intensities were seen radially. The TLs and TALs of the loops of Henle had a positive cytoplasm, but the DCTs showed negative or faint labeling. OMCDs and IMCDs showed positive labeling in the cytoplasm, but CCDs showed negative or faint labeling.

**Figure 6:**
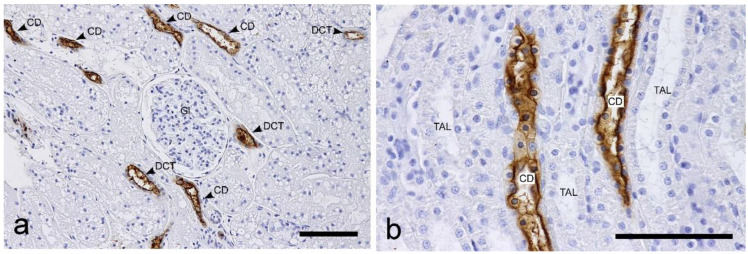
Histochemical staining for Peanut agglutinin (PNA) in the feline kidney. (**a**) Low magnification view of the cortex. (**b**) High magnification view of the inner medulla. Positive labelings are strong in the CDs and DCTs. Scale bars: 100 μm.

PNA: Strong labeling was observed in the TLs, DCTs, and CDs, but not in the TALs of the loops of Henle (Figure 6). Labeling was also focused on the apical surfaces. No labeling was observed in the glomeruli or PTs.

**Figure 7:**
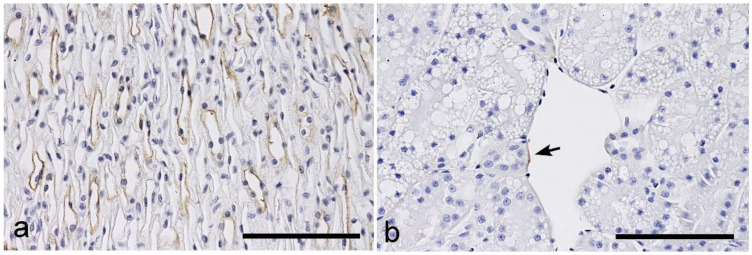
Histochemical staining for *Soybean* agglutinin (SBA) and *Dolichos biflorus* agglutinin (DBA) in the feline kidney. (**a**) SBA: inner medulla. Positive labeling can be observed in the thin limbs of the loops of Henle. (**b**) DBA: superficial area of the cortex. Positive labeling can be regionally observed in the vascular endothelium of a capsular vein (arrow). Scale bars: 100 μm.

SBA: No labeling was observed in the glomeruli or PTs. In the loops of Henle, the TLs showed strong labeling (Figure 7a), while the TALs showed negative or faint labeling. The CDs also exhibited negative or faint labeling.

DBA: No labeling was observed in the nephron segments from the glomeruli to the CDs, but rare positive endothelial cells were detected, especially in the capsular veins (Figure 7b).

**Figure 8:**
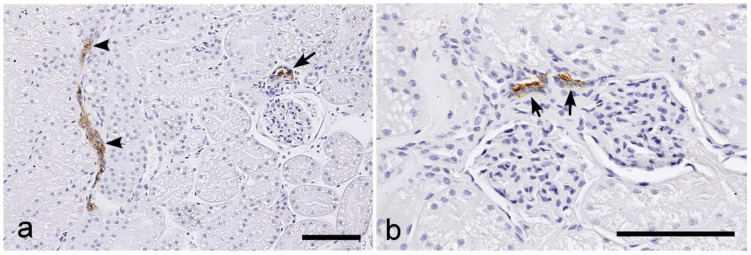
Histochemical staining for *Ulex europaeus* agglutinin I (UEA-I) in the feline kidney. (**a**) Low magnification view of the cortex. (**b**) High magnification view of the cortex. Positive labeling can be observed in the distal tubules, especially in the macula densa regions (arrows). A few DCTs also show positive labeling (arrowheads). Scale bars: 100 μm.

UEA-I: Positive labeling was sparsely observed in the TALs of the loops of Henle and DCTs (Figure 8a), in close proximity to the glomeruli and the macula densa regions, which showed strong labeling (Figure 8b). No positive labeling was observed in other parts of the nephron segments.

## 4. Discussion

In this study, the binding patterns of eight types of lectins (WGA, s-WGA, ConA, RCA-I, SBA, PNA, BDA, and UEA-I) in the nephron segments of normal feline kidneys was assessed. Regarding the glomeruli, the glomerular capillary wall (GCW) was labeled by WGA; however, this was not considered a feline-specific feature as the same labeling has been confirmed in various species, including dogs [20,33]. Although WGA is a lectin that binds to N-acetylglucosamine, it also reacts with sialic acid residues [32]. Since N-acetylglucosamine-binding s-WGA showed no positive labeling in the GCW, the labeling of WGA implies the presence of sialic acid residues in the GCW. This finding for GCW was shown not only in cats in the present study, but also in the dogs in our previous report [20]. Sialic acid is known as an essential carbohydrate chain for maintaining the filtration barrier of the GCW [34]. In human kidneys, a lectin histochemical study, using sialic acid-specific lectins, such as *Tritrichomonas mobilensis*, *Maackia amurensis II*, and *Sambucus nigra*, suggested that sialyation of the glycoprotein in glomeruli is altered in kidney diseases, its changes, though, were not specific to the specific type of kidney disease [15]. However, changes in sialylation in the glomeruli in kidney diseases of dogs and cats have not been previously demonstrated, and should be investigated in a future study.

In our previous survey of normal canine kidneys, GCW was shown to be heterogeneously labeled with RCA-I [20]. In the analysis of the ICGN of dogs, a reduction of this RCA-I labeling was found in 6 out of 13 cases, despite no changes in WGA labeling in the GCW [21]. In the present study, such labeling by RCA-I in the GCW was not observed in feline kidneys. RCA-I is a lectin that binds to galactose residues. In a previous histochemical study investigating lectin binding in rat kidneys, lectins binding to beta-galactose residues prominently bound to the glomerular basement membrane in fetal rats, but this reactivity decreased with postnatal development [35]. Although the physiological significance of RCA-I labeling in the GCW remains unclear, this difference may reflect the difference in maturity of the investigated glomeruli between dogs and cats.

In PTs, clear positive labeling was detected for WGA, s-WGA, RCA-I, and ConA lectins, but not for other lectins. Although this binding pattern was essentially the same as that of canine kidneys [20], the feline kidneys showed interesting heterogeneity in labeling intensity. Briefly, areas with strong and weak labeling were mixed and radially arranged within the same section, and this heterogeneity was more prominent in PCTs than in PSTs within the renal cortex. In the present study, heterogenicity was found in all four cats, but this differentiation of compartments was not observed in HE, PAS, and MT stains. Although there have been no reports regarding the functional meanings of this heterogeneity, unknown functional compartments that have not been clarified may be present in the feline kidney. Based on the sugar specificity of the lectins (Table 1), it is possible that glycosylation with various carbohydrates, such as N-acetylglucosamine, α-mannose, α-D-glucose, and galactose, is related to the functional differentiation of cortical compartments. In addition, the basement membrane of the PTs was labeled with WGA and RCA-I. In particular, the labeling for WGA was clear but showed heterogeneity across areas. Since s-WGA showed no positive labeling at this site, this labeling for WGA might imply the heterogeneous presence of sialic acid residues in the basement membrane of the PTs in the feline kidney. This site functionally maintains the mineral and acid–base balances of the body via various channels and pumps, and the feline kidney might have the heterogeneity of this function in the PTs.

PNA is a lectin that binds to *galactosyl* (*β*-1, 3) N-*acetylgalactosamine* (*Galβ3GalNAc*), and this lectin was specifically bound to the distal nephrons, other than the TALs of the loops of Henle; these findings were similar to those in dogs [20]. Studies on human kidneys have demonstrated that PNA is a lectin that binds to distal nephrons [5,33]. However, a comparative study, investigating the kidneys of 14 animal species, showed that the binding sites of the PNA were apparently different between species, and some species showed positive labeling in the distal nephrons, as in humans, while others showed positive labeling in the proximal nephrons [33]. Although the physiological roles reflected in the species-dependent differences in PNA, binding in the nephron segment, have not yet been clarified, it was demonstrated that cats belonged to the animal group with kidneys with PNA-positive distal nephrons.

DBA and SBA are lectins which bind specifically to acetylgalactosamine; labeling for these lectins was absent or faint in the nephron segments. No positive labeling for DBA was detected in any segment of the nephron. Interestingly, among 14 investigated animal species (elephant, cow, goat, pig, dog, rabbit, guinea-pig, rat, mouse, hen, quail, frog, trout, and man), similar findings have only been reported in the trout [33]. Some lectins have been used not only as markers of residual carbohydrates, but also as functional or pathophysiological markers, and the potential of DBA lectin as an indicator of cell differentiation and morphogenesis has been previously reported [36,37]. The species-dependent difference in the DBA-binding pattern might be related to that in cell differentiation and the urine concentrating ability of the distal nephron; however, we could not find any related evidence in the literature.

Regarding s-WGA, heterogeneity was remarkable in the CDs, comprising cells with positive cytoplasm and those with negative cytoplasm. A heterogeneous lectin-binding pattern in CDs has been reported in humans [3], rats [38], mice [31], and dogs [20]. It is well known that the mammalian collecting duct consists of principal and intercalated cells (ICs). In the present analysis, the s-WGA-positive cells accounted for a minority proportion of OMCD cells, and were scarce in the IMCD cells, and such a distribution of the cells corresponded with that of the ICs. This finding was the same as that in a previous report on the canine kidney [20]. There are three types of ICs: A-ICs, B-ICs, and non-A non-B-ICs [39], which all contribute to the regulation of acid-base balance and electrolyte homeostasis [39,40]. The disturbance of the acid-base balance and electrolyte homeostasis, such as in metabolic acidosis, hypokalemia, and hypochloremia, are frequently experienced in feline CKD, and dysfunction of the ICs may correspond with the progression of the disease. The morphology and physiology of ICs have not been fully clarified in canine and feline kidneys, and it has been proposed that s-WGA lectin might have potential as a probe to investigate the pathophysiological changes of ICs in feline CKD. This potential of the s-WGA may be related to its high specificity for N-acetylglucosamine. In WGA, which also binds to N-acetylglucosamine, broad rather than heterogeneous labeling was observed throughout the CDs, and it is known that WGA can also react with other sugar moieties, such as sialic acids [32]. Therefore, the s-WGA-positive heterogeneous labeling in CDs might imply the heterogeneous localization of the N-acetylglucosamine residues.

Positive UAE-I labeling was observed in the distal tubules, particularly in the macula densa. A similar binding pattern for UEA-I has also been demonstrated in normal canine kidneys [20]. In a previous analysis regarding the ICGN of dogs, some showed expansion of the UEA-I-binding sites, while others showed a reduction in labeling [21]. The macula densa is a special region in the TALs of the loops of the nephron, and contributes to controlling fluid balance and blood pressure as a sensor for the tubular feedback system and the renin-angiotensin system. The specific binding to this site of the nephron is interesting, and it is expected that UEA-I might have the potential to be used as a probe to investigate the pathophysiological changes of the macula densa in canine and feline kidney diseases. UEA-I is a lectin specific for fucose. In the macula densa region, positive labeling of this lectin was observed in the apical surface of cells, and it was suggested that fucosylation of the glycoprotein might be essential for its cellular function as a sensor of the luminal fluid composition in the macula densa.

## 5. Conclusions

The present study surveyed the binding patterns of eight lectins with commercially available detection kits to nephron segments in normal feline kidneys. Some of the binding patterns demonstrated here were different from those in the kidneys of other mammals, including dogs [20,33], and showed some feline-specific lectin binding patterns. Changes in the renal residual sugar composition on cell surfaces and intracellular organelles should correlate with the pathophysiological mechanisms of kidney diseases, and the present findings will be important in future analyses targeting changes in glycosylation in feline kidney diseases.

## Figures and Tables

**Table 1:** Lectins used in this study.

Lectin	Concentration	Sugar/Rough Sugar Specificity	Hapten Sugar Concentration
Wheat germ agglutin (WGA)	4 μg/mL	GlcNAc/βGlcNAc, sialic acid, GalNAc	360 mM GlcNAc
Succinylated wheat germ agglutin (s-WGA)	8 μg/mL	GlcNAc	360 mM GlcNAc
*Ricinus communis* agglutin I (RCA-I)	4 μg/mL	Gal	180 mM Gal
Concanavalin A (ConA)	4 μg/mL	αMan, αGlu/branched and terminal Man	180 mM α-methyl mannoside and glucoside
*Peanut* agglutinin (PNA)	8 μg/mL	Galβ3GalNAc/terminal Gal	180 mM Gal
Soybean agglutinin (SBA)	8 μg/mL	α > βGalNAc/α or β-terminal GalNAC, α1, 3Gal	90 mM GalNAc
*Dolichos biflorus* agglutinin (DBA)	8 μg/mL	αGalNAc/Galβ1, 4GlcNAc	90 mM GalNAc
*Ulex europaeus* agglutinin I (UEA-I)	8 μg/mL	αFuc	90 mM Fuc

The sugar specificity of lectins is based on the manufacture’s guidelines [32]. Fuc: L-Fucose, Gal: D-Galactose, GalNAc: N-Acetylgalactosamine, Glu: D-Glucose, GlcNAc: N-Acetylglucosamine, Man: Mannose.

**Table 2:** Lectin binding patterns in feline kidneys.

	WGA	s-WGA	RCA-I	ConA	PNA	SBA	DBA	UEA-I
**Gl**								
Cap	H	−	−	−	−	−	−	−
BC	H	−	H	H	−	−	−	−
Mes	++/−	−	−	−	−	−	−	−
**PCT**								
Cy	H	−	−/+	H	−	−	−	−
BB	H	H	+	H	−	−	−	−
BM	H	−	−	−	−	−	−	−
**PST**								
Cy	H	−	−/+	H	−	−	−	−
BB	+	++	+++	H	−	−	−	−
BM	H	−	−/+	−	−	−	−	−
**TL**	++	++	++++	+++	++	+++	−	−
**TAL**								
AS	+++	++	−	−	−	−	−	+/++
Cy	++	−/+	−	−/+	−	−/+	−	+/++
**DCT**								
AS	++	H	+++	−	+++	−/+	−	+/++
Cy	+	+	++	−/+	+	−/+	−	+/++
**CCD**								
AS	+++	++	+++	−/+	+++	−	−	−
Cy	++	H*	++	−/+	+	−/+	−	−
**OMCD**								
AS	+++	−	+++	−/+	+++	−	−	−
Cy	++	H*	++	+	+	−/+	−	−
**IMCD**								
AS	+++	+++	+++	+	+++	−/+	−	−
Cy	++	++	++	+	+	−/+	−	−

Gl: glomeruli, Cap: capillary, BC: Bowman’s capsule, Mes: mesangium, PCT: proximal convoluted tubules, Cy: cytoplasm, BB: brush border, BM: basement membrane, PST: proximal straight tubules, TL: thin limbs of the loop of Henle, TAL: thick ascending limbs of the loop of Henle, AS: apical surface, DCT: distal convoluted tubules, CCD: cortical collecting ducts, OMCD: outer medullary collecting ducts, IMCD: inner medullary collecting. H: heterogeneity; staining intensity is different between areas, and areas with strong labeling and those with weak labeling were mixed within the same section. H*: heterogeneity; staining intensity is different between the cells within the same duct. −: negative, +: weak, ++: moderate, +++: strong.

## Data Availability

The data are available on request from the corresponding author.
